# Supplementary material for: Investigating the impact of HIV on patients with first episode psychosis: a study protocol for a longitudinal cohort study
Source: BMJ Open. 2021 May 21;11(5):e046593. doi: 10.1136/bmjopen-2020-046593 (PMC8144045; doi:10.1136/bmjopen-2020-046593)
Supplement: Author's manuscript [file bmjopen-2020-046593.draft_revisions.pdf]

BMJ Open is committed to open peer review. As part of this commitment we make the peer review history of every article we publish publicly available.

When an article is published we post the peer reviewers' comments and the authors' responses online. We also post the versions of the paper that were used during peer review. These are the versions that the peer review comments apply to.

The versions of the paper that follow are the versions that were submitted during the peer review process. They are not the versions of record or the final published versions. They should not be cited or distributed as the published version of this manuscript.

BMJ Open is an open access journal and the full, final, typeset and author-corrected version of record of the manuscript is available on our site with no access controls, subscription charges or pay-per-view fees (http://bmjopen.bmj.com).

If you have any questions on BMJ Open's open peer review process please email info.bmjopen@bmj.com

Investigating the impact of HIV on patients with first episode psychosis: a study protocol for a longitudinal cohort study

Journal:	*BMJ Open*
Manuscript ID	bmjopen-2020-046593
Article Type:	Protocol
Date Submitted by the Author:	04-Nov-2020
Complete List of Authors:	Chhagan, Usha; University of KwaZulu-Natal,<br>Ntlantsana, Vuyokazi; University of KwaZulu-Natal<br>Tomita, Andrew; University of KwaZulu-Natal College of Health Sciences,<br>Naidu, Thirusha; University of KwaZulu-Natal<br>Chiliza, Bonginkosi; University of KwaZulu-Natal, School of Clinical Medicine<br>Paruk, Saeeda; University of KwaZulu-Natal
Keywords:	Schizophrenia & psychotic disorders < PSYCHIATRY, HIV & AIDS < INFECTIOUS DISEASES, MENTAL HEALTH

SCHOLARONE™
Manuscripts

I, the Submitting Author has the right to grant and does grant on behalf of all authors of the Work (as defined in the below author licence), an exclusive licence and/or a non-exclusive licence for contributions from authors who are: i) UK Crown employees; ii) where BMJ has agreed a CC-BY licence shall apply, and/or iii) in accordance with the terms applicable for US Federal Government officers or employees acting as part of their official duties; on a worldwide, perpetual, irrevocable, royalty-free basis to BMJ Publishing Group Ltd ("BMJ") its licensees and where the relevant Journal is co-owned by BMJ to the co-owners of the Journal, to publish the Work in this journal and any other BMJ products and to exploit all rights, as set out in our licence.

The Submitting Author accepts and understands that any supply made under these terms is made by BMJ to the Submitting Author unless you are acting as an employee on behalf of your employer or a postgraduate student of an affiliated institution which is paying any applicable article publishing charge ("APC") for Open Access articles. Where the Submitting Author wishes to make the Work available on an Open Access basis (and intends to pay the relevant APC), the terms of reuse of such Open Access shall be governed by a Creative Commons licence – details of these licences and which Creative Commons licence will apply to this Work are set out in our licence referred to above.

Other than as permitted in any relevant BMJ Author's Self Archiving Policies, I confirm this Work has not been accepted for publication elsewhere, is not being considered for publication elsewhere and does not duplicate material already published. I confirm all authors consent to publication of this Work and authorise the granting of this licence.

For peer review only

Investigating the impact of HIV on patients with first episode psychosis: a study protocol for a longitudinal cohort study

Usha Chhagan[1], Vuyokazi Ntlantsana[1], Andrew Tomita[2,3], Thirusha Naidu[4], Bonga Chiliza[1], Saeeda Paruk[1]

[1] Discipline of Psychiatry, Nelson R Mandela School of Medicine, University of KwaZulu-Natal, Durban, South Africa.

[2] KwaZulu-Natal Research Innovation and Sequencing Platform (KRISP), College of Health Sciences, University of KwaZulu-Natal, Durban, South Africa

[3] Centre for Rural Health, School of Nursing and Public Health, College of Health Sciences, University of KwaZulu-Natal, Durban, South Africa

[4] Department of Behavioural Medicine, University of KwaZulu-Natal, Durban, South Africa

Corresponding author
Dr Usha Chhagan

Author affiliations
Usha Chhagan, uchhagan@hotmail.com, Discipline of Psychiatry, Nelson R Mandela School of Medicine, University of KwaZulu-Natal, Durban, South Africa. +27 031-260-4321.

Vuyokazi Ntlantsana, Ntlantsanav@ukzn.ac.za, Discipline of Psychiatry, Nelson R Mandela School of Medicine, University of KwaZulu-Natal, Durban, South Africa. +27 031-260-4321.

Andrew Tomita, Tomita@ukzn.ac.za, KwaZulu-Natal Research Innovation and Sequencing Platform (KRISP), College of Health Sciences, University of KwaZulu-Natal, Durban, South Africa. Centre for Rural Health, School of Nursing and Public Health, College of Health Sciences, University of KwaZulu-Natal, Durban, South Africa. +27 031-260-4321.

Thirusha Naidu, naidut10@ukzn.ac.za, Department of Behavioural Medicine, University of KwaZulu-Natal, Durban, South Africa. +27 031-260-4321.

Bonga Chiliza, ChilizaB@ukzn.ac.za, Discipline of Psychiatry, Nelson R Mandela School of Medicine, University of KwaZulu-Natal, Durban, South Africa.

Saeeda Paruk, saeedaparuk@gmail.com, Discipline of Psychiatry, Nelson R Mandela School of Medicine, University of KwaZulu-Natal, Durban, South Africa. +27 031-260-4321.

For peer review only

Authors' contributions
UC, VN, TN and SP conceived the study. UC, VN will collect data. AT performed the statistical analysis. SP and BC supervised the work undertaken. All authors have read and approved the final manuscript.

Funding statement
This study is funded by National Research Foundation of South Africa (Grant #: 117858) and the Society for Biological Psychiatry Research Fund. Funding (Grant #: N/A) was also received from University of KwaZulu-Natal College of Health Sciences PhD Scholarship (Grant #: N/A). The opinions and findings presented are those of the authors and not a reflection of the funders.

Competing interests
None declared.

For peer review only - http://bmjopen.bmj.com/site/about/guidelines.xhtml

Investigating the impact of HIV on patients with first episode psychosis: a study protocol for a longitudinal cohort study

ABSTRACT

Introduction: South Africa has a high HIV prevalence and limited mental health care resources. Neuropsychiatric complications such as psychosis onset in people living with HIV (PLWHIV) remains poorly understood. The study aims to compare the socio-demographic, clinical, substance use, cognitive and trauma profile of PLWHIV presenting with first episode psychosis (FEP) to those with the condition but without HIV.

Methods and Analysis: This study will compare presentation, course, and outcome of a cohort of PLWHIV and FEP with a control group recruited over a 3-year period. We will prospectively test the hypothesis that the 2 groups are socio-demographically, clinically and cognitively distinct at illness presentation, with higher trauma burden and poorer outcomes in those with the dual burden of HIV and FEP. FEP participants, confirmed by a structured neuropsychiatric interview, will have their socio-demographic, psychosis, mood, motor, trauma, and substance use variables assessed. A neuropsychological battery will be completed to assess cognition, while quality of life, psychotic symptoms and HIV markers will be measured at 3, 6 and 12 months.

Ethics and dissemination: The study protocol has been reviewed and ethics approval obtained from the Biomedical Research Ethics Committee (BC 571/18) of the University of KwaZulu-Natal. The results from this investigation will be actively disseminated through peer-reviewed journal publications and conference presentations.

Keywords: Mental Health; HIV; Psychosis; Africa.

For peer review only

Strengths and limitations of this study

- The study may provide valuable insight into the long-term impact of HIV infection on psychosis presentation which is poorly understood in low- and middle-income countries.
- Extensive neuropsychological assessments will be undertaken.
- Randomized controlled trial (RCT) design is not possible given that study monitors disease progression.

For peer review only

For peer review only - http://bmjopen.bmj.com/site/about/guidelines.xhtml

INTRODUCTION

Psychiatric illness in the presence of Human Immunodeficiency virus (HIV) and Acquired Immunodeficiency Syndrome (AIDS) has been linked to negative health behaviours and poorer clinical outcomes[1]. The prevalence of HIV among those with serious mental illness (SMI) ranges from 1 to 24%[2] . Left untreated, mental disorders in people living with HIV (PLWHIV) result in poorer quality of life, greater interpersonal difficulties, increased suicide risk and poor adherence to antiretroviral therapy.

Psychotic disorders are associated with a significant burden of disease, with affected persons often relying on limited public sector mental health resources in low- and middle-income countries (LMICs)[3]. There is a lack of research on HIV and psychosis in LMICs, particularly sub-Saharan Africa (SSA), despite this region accounting for more than two-thirds of the world's HIV-positive population[4]. While research from high-income countries has demonstrated that there is a bi-directional relationship between HIV/AIDS and mental illness, there may be differences in the way this relationship is expressed in SSA[5]. Studies have also shown variations in the socio-demographic and clinical profile of those with dual burden of HIV and first episode psychosis compared to those with psychosis only[6].

Socio-demographic profile of PLWHIV and first episode psychosis

There is limited literature on the association of HIV and first episode psychosis (FEP), with previous studies mainly describing HIV prevalence rates or clinical profiles[7]. A Ugandan study by Lundberg and colleagues[8] found that HIV positive patients with mental illness were more likely to be females and older (40-49 years). A KwaZulu-Natal (KZN) Province, SA, study of patients with FEP reported a higher HIV prevalence in less educated individuals, however, this was a small sample[7]. In a more recent chart review of patients with psychosis admitted to a psychiatric hospital in SA, HIV-infected patients with psychotic disorders were more likely to be female (74.0%), younger than 50 years (94.0%) and less likely to have secondary education than HIV-negative patients with psychotic disorders (56.0% vs. 72.0%)[9].

PLWHIV and psychosis symptomatology

In a review of the literature, grandiose, persecutory, and somatic delusions were the most common psychotic symptoms, followed by hallucinations and mood symptoms in PLWHIV

For peer review only - http://bmjopen.bmj.com/site/about/guidelines.xhtml

with FEP[10]. An Italian study found that HIV infection increased the severity of symptoms (more frequently paranoid delusions), was associated with a greater impairment in attention and concentration, and had decreased depressive symptoms[6]. In addition, PLWHIV and psychosis are more likely to be diagnosed as having a psychosis secondary to another medical condition than a primary psychotic disorder, have more comorbid medical disorders and increased haematological test abnormalities[9]. However, there is extremely limited literature on HIV and psychosis symptomatology, particularly from Africa[7,11]. This suggests a hiatus in the current literature on understanding the nature and severity of psychotic symptoms in PLWHIV, particularly in the African context. There is a need for systematic studies of larger cohorts with standardised tools to better understand how HIV may potentially influence psychosis presentation and course, as well as HIV disease outcomes.

PLWHIV and FEP and cognition

Cognitive impairment is well established in both psychotic disorders and HIV infection, being exacerbated in psychotic patients with HIV[11]. Being HIV positive is traditionally associated with a sub-cortical cognitive impairment, the prevalence of cognitive deficits being reported to range from 4% - 99% in a systematic review of HIV research in SSA[5].

De Ronchi[6] described cognitive screen deficits in a sample of 22 FEP patients living with HIV in Italy. Participants had impaired attention and concentration on the Mini Mental State Examination (MMSE) but no other differences on cognitive impairment. A study of cognitive dysfunction among 156 HIV infected and 322 HIV non-infected patients with psychosis in Uganda assessed them with the MMSE and a neuropsychological battery. They found that PLWHIV and psychosis were more cognitively impaired than HIV negative psychotic patients at baseline, and although there was some improvement with treatment, they remained more impaired at six months, suggesting that HIV worsened cognitive dysfunction in psychosis[11]. While earlier studies have suggested that psychosis and impairment in cognition were later manifestations of HIV, the Ugandan study demonstrated that both these conditions occur early, as shown by the average CD4 count of 305 cell/uL and the intermediate World Health Organization (WHO) staging of disease presentation. In addition, impairment in cognition also remained, despite an improvement in the psychotic symptoms[11]. Further research is warranted to establish the impact of HIV and psychosis on

For peer review only - http://bmjopen.bmj.com/site/about/guidelines.xhtml

cognition, and whether HIV related cognitive changes are associated with increased risk for psychosis or modifies psychosis onset or course.

PLWHIV and FEP and substance use

Studies traditionally suggest an association between substance use in FEP patients and HIV infection[1]. A study of patients with FEP in KwaZulu-Natal (KZN), SA reported lower rates of self-reported substance use among HIV positive then HIV negative patients with mental illness[7], which was further supported by a retrospective chart review of patients with psychosis[9]. This may suggest that either substance use/misuse may not be a significant factor in HIV transmission in the KZN context, or that the study results were limited by under-reporting or the small sample size. Thus, while the international literature suggests that PLWHIV and mental illness may be vulnerable to substance use, this has not been borne out in the local KZN studies, and warrants further research.

PLWHIV and FEP and Trauma

Trauma, particularly early life trauma, is also recognised as an environmental risk factor for psychosis[12]. A South African study investigated the association between a history of traumatic experiences and the clinical features of FEP. Duration of untreated psychosis, age of and symptoms at onset were assessed in 54 psychotic patients, while 22% were HIV infected. Almost half of the study subjects had witnessed (49%) or directly experienced serious physical assault (45%), both events being associated with positive psychotic symptoms[13].

The trauma experienced may influence psychosis and HIV outcomes, as found in a systematic review, that hallucinations and delusions were more severe in people with a history of childhood trauma[14]. In addition, Leserman[15] reported on the negative association of HIV, stress, and depression on the course of HIV in terms of decreasing the CD4 lymphocytes, increasing the viral load and having greater risk for premature mortality.

Course of comorbid HIV and psychosis

PLWHIV and psychosis are more likely to develop comorbid medical disorders, experience side effects of antipsychotic medication, develop metabolic syndrome secondary to ART, have longer length of hospital stay and readmission rates, and poorer quality of life[1,9].

For peer review only - http://bmjopen.bmj.com/site/about/guidelines.xhtml

However, this has not been tested prospectively in the FEP patients in the African context with the recent wide availability of ART.

Aims

The aim of the study is to investigate trends in clinical, substance use, cognitive and trauma presentations in individuals experiencing first episode psychosis (FEP) over time, and to compare presentation, course and 12-month outcomes between those with and without HIV.

METHODS

Patients referred to the participating psychiatry units will be screened by the respective treating doctors for eligibility for recruitment into the study. The patients with first episode of psychosis must fulfil the inclusion criteria and will be referred to the designated investigator at each unit. Once written informed consent has been obtained, the protocol for data capture will be followed, as per the list outlined in Table 1 and Figure 1, the same protocol being followed for out- and in-patients. The clinical examination and psychiatric tests, which take approximately two hours, will be undertaken by the investigators who are all medical practitioners. Patient baseline assessments will be undertaken within six weeks of treatment during their outpatient visit or following admission to the respective units at baseline. A physical examination, body mass index and waist circumference measure will also be obtained by a medical practitioner. Where possible, collateral information from a next of kin, with the participant's consent, will also be sought. The follow-up visits will be done at three, six and 12 months as outpatients, and the participants will be reimbursed for travel costs. Attempts will be made to coincide each of these with scheduled clinic follow-up visits and will be performed by the same initial interviewer at each psychiatry outpatient department.

The cognitive battery assessment, which takes approximately 2 hours, will be administered by a clinical psychologist, at the three- and 12-month visits. Translation services will also be available for patients who require isiZulu translation during the interviews.

Following collating the data for steps 1-13 in Figure 1 and Table 2, we will perform steps 14 and 15 after voluntary counselling and testing. The required HIV tests, CD4 count and viral load if HIV positive, as per the protocol, will be taken by a medical practitioner at each site.

For peer review only - http://bmjopen.bmj.com/site/about/guidelines.xhtml

Other investigations, such as lipid profile, which are part of routine care, will be captured from the clinical records.

Study design and setting

The comparative cohort study will be in keeping with a quantitative, descriptive and longitudinal 12-month design of adult patients presenting with FEP that are either HIV infected or non-infected. The study will take place in the eThekwini District, one of 11 in KZN Province, with an estimated population of 3,442,361 people. The district has a high HIV burden, with an estimated 650 000 people living with the virus in 2018[16]. In addition, there are only five hospitals in the municipality with psychiatric services managed by a psychiatrist, at which the study will be conducted. The hospitals selected are the four general hospitals with specialist psychiatric services situated in eThekwini: Addington, King Edward VIII, Prince Mshiyeni and RK Khan Hospitals, and at King DinuZulu Hospital, which serves as the psychiatric hospital for the area. All hospitals have in- and out-patient psychiatric services and receive referrals from within the district as well as from urban and rural areas as far south as the Eastern Cape Province and northern KZN.

Male and female patients will be included if they are in or out-patients, aged 18-45 years, have a first presentation to mental health care services for FEP meeting Diagnostic and Statistical Manual (DSM)-V criteria[17] (American Psychiatric Association (APA) 2013) for a psychotic disorder, are neuroleptic naïve or minimally treated (maximum 6 weeks of psychotropic treatment in this first episode), and provide consent to participate.

Patients will be excluded if they have been prescribed antipsychotics in the past or have had previous psychotic episode, an intellectual disability (assessed clinically or by chart review) or other significant general medical conditions that maybe associated with FEP e.g. epilepsy or syphilis, have less than seven years of formal schooling and refuse consent.

Participant selection and sampling strategy

All adult in-or out patients with FEP meeting the inclusion criteria will be recruited to the study over a 3-year period. The sample size calculation was based on a log rank test group. In order to achieve an 80% power to detect an odds ratio of 1.857 (to maximise variability) in a design with three repeated measurements having a compound symmetry covariance structure

For peer review only - http://bmjopen.bmj.com/site/about/guidelines.xhtml

when the proportion from group 2 is 0.500, the correlation between observations on the same subject is 0.500, and the alpha (significance) level 0.05 or 5%. In addition, to allow for and give an expected 36-month accrual, a minimum follow-up of 12 months, and a 10% dropout rate, a sample of 130 PLWHIV and FEP and 130 HIV negative with FEP will be required.

Data collection

The study will utilize a clinical interview, physical examination, several psychiatric tools, and HIV related haematological tests to measure variables. Baseline measures will be done within six weeks of first presentation and as soon as the participant is able to consent. Participants need to have responded to antipsychotic medication and will be assessed to have the capacity to consent by a treating and research doctor. The study (HIV+) and control groups (HIV-) will receive the same baseline assessments and follow-up at three, six and 12 months, which are summarised in Table 1. The HIV negative group will have HIV testing at each visit to re-confirm HIV status. The study procedures that will be followed are shown in the information box 1. Data will be collected manually and captured electronically.

Clinical researchers with a medical background will administer the mental health diagnostic -assessment, the MINI 7.02, Psychotic Disorders version, DSM-V[18] and diagnostic interview face-to-face, and will be supported by a trained research assistant who will facilitate the self-report questionnaires. A medical doctor will conduct the clinical assessments, collect the biological specimens (Table 1), and conduct the HIV counselling and testing. We will use an HIV COBAS COMBI test to detect both HIV antibodies and p24 antigen. Standard pre-test and post-test counselling will be delivered in a culturally sensitive manner, while the CD4 count and viral loads will be measured at every visit among PLWHIV.

Participants will be given a return research appointment date and telephonic contact will be maintained between interviews. The study team (research assistant and clinician) will contact participants to remind them of appointments to limit their withdrawing from the study. For participants who do not arrive for their appointments, the research assistant will contact the patient to reschedule an appointment in the same week.

For peer review only - http://bmjopen.bmj.com/site/about/guidelines.xhtml

Data management and analysis

All participants will be allocated a participant identification number (PID) code at enrolment, which will be used on samples and documents over the 12-month study period. Data will be downloaded to a secure server and kept in a password-protected system. Laboratory results will be merged into the main dataset using the unique PID.

Data was will be entered into SPSS version 24 and the analyses conducted using STATA 15. The socio-demographic and clinical characteristics of participants will be summarized using means (and standard deviation (SD) continuous variables. For categorical variables, proportion (%) will be reported. Significant associations in contingency tables (cross tabulations) will be assessed using the standard Pearson's chi-square (χ^2) test. An independent samples t-test will be used for comparing differences in continuous variables between two groups. If there are more than three groups, we will utilise ANOVA methods. A p-value less than 0.05 will be regarded as statistically significant.

Patient and public involvement

No patients or members of the public were involved in the design, analysis or reporting of this current investigation.

Ethics and dissemination

Ethical approval to conduct the study was obtained from the Biomedical Research Ethics Committee of the University of KwaZulu-Natal (UKZN). The study will be conducted in accordance with South African Department of Health Research Ethics guidelines (2015) as well as the UKZN policy on Research Ethics. Recruitment of study participants will commence as full ethics approval was received from Biomedical Research Ethics Committee. No other independent ethics review was required.

All participants fulfilling the inclusion criteria will be selected for the study, from whom written, informed consent will be obtained. All potential participants will have the opportunity to participate, which will be made known to them in the information sheet. Where participants are unable to consent independently, adequate steps will be taken to ensure that legally acceptable proxy consent is obtained. All participants testing HIV positive at any point in the study will be referred for ART and support.

For peer review only

The results from this investigation will be actively disseminated through peer-reviewed journal publications and conference presentations. Upon completion of the peer-review process, we will provide feedback to clinicians at Department of Health for further discussion about enhanced treatment algorithm.

DISCUSSION

Potential risks

There will be minimal risk or discomfort to participants, with one test requiring bloods to be taken. The only active involvement of the participants is for them to provide verbal responses to assessment interviews and scales utilized for data collection. We acknowledge the potential risk of psychological distress to patients, as they may be directly affected by sensitive questions e.g. relating to trauma during the administration of the psychiatric scales. The study has a distress plan to support such patients, which entails referral to treating team if there is concern about relapse or danger to self or others.

Methodological challenges and study limitations

Several limitations may affect the study, including that it is hospital-based, which may introduce sample bias and limit the generalizability to community samples. Another limitation is the use of tools that have limited validity in the local South African context, as they have not been used previously. Language and cultural barriers in communication will be addressed by an available isiZulu translator. A further limitation of the observational nature of the study is the attendant lack of standardisation of treatment, for which will follow the Essential Drug List (EDL) for hospital level adults (Department of Health, 2015). Although patients will follow the same algorithm, they may be maintained on different medication, depending on the individual patient treatment response. The study will however provide an adequate sample representing the clinical profile of patients with FEP and LWHIV in a resource-constrained setting.

Study progress and challenges

A challenge we anticipate is the loss of participant follow-up, which is generally a common failing in managing psychiatric patients, particularly in LMIC settings with challenging psychosocial circumstances. The three-, six- and 12-month follow-up appointments may

For peer review only - http://bmjopen.bmj.com/site/about/guidelines.xhtml

remain difficult to sustain, despite supporting participants with travel costs for visits. Maintaining follow-up visits is a challenge we anticipated and for which we have a contingency strategy, this being that a research assistant will have regular communication with the recruited participants, confirming appointment dates, sending reminders, and facilitating travel arrangements. Participants seroconverting to HIV positive status during the study will be referred to antiretroviral services for care but continue follow-up assessments, noting the change in their HIV status.

Study Significance

We postulate that the course and outcome of HIV psychosis differs from that in an HIV negative individual, and that this study may provide valuable insight into the impact of HIV infection on psychosis presentation and outcomes. Longitudinal comparative cohort studies of this nature are limited and have not been conducted locally in one of the highest HIV prevalence settings in Africa, hence the data will serve to introduce new knowledge on the topic, as well as form the baseline for future research and treatment. A clearer understanding of the association between HIV and psychosis may provide better insight into HIV as a neurotropic virus. The findings of this study will contribute towards improving the care and management of patients with FEP and HIV.

The World Health Organization reports that in 2020, psychiatric conditions are ranked the second leading cause of global disease burden after ischemic heart disease[19]. In the South African context, this is even more pronounced by the additional burden of HIV infection and AIDS. With an approximate overall HIV prevalence rate of 11.2% (6.19 million) and an estimated 16.6% of the population (adults aged 15-49 years) being HIV positive, South Africa is in urgent need of interventions to address the related adverse consequences. Psychotic disorders are associated with a significant burden of disease and often utilize the limited mental health resources. Understanding the association between HIV and psychosis in the era of antiretroviral treatment is thus an urgent priority. Utilizing the HIV psychosis as a potential model for schizophrenia may also improve our understanding of its neurobiology. The new knowledge, generated through this novel study, will enable an understanding of the interplay between HIV and psychosis at a fundamental level, centred at the international epicentre of the HIV pandemic.

For peer review only - http://bmjopen.bmj.com/site/about/guidelines.xhtml

In addition, in South Africa, substance use and trauma remain critical public health challenges that need to be further explored. Thus, the study of the bidirectional associations between HIV, substance use and traumatic experiences, psychosis onset and presentation in a low- and middle-income setting is essential to better guide the strategic use of their limited resources. Early insights into the role of substance use and trauma in HIV infection and psychosis onset could be used to delay, modify, or even prevent the onset of HIV psychosis. Furthermore, the interplay of these extraneous factors in genetically predisposed individuals has not been explored. This study will allow us to begin to understand the contribution of HIV to psychosis onset, increase awareness of the need for screening and care in a highly vulnerable group, while building research capacity.

The results of this study will contribute to the development of management (investigation and treatment) algorithms in the South African resource constrained health departments. Reference will be made to the differences identified between the HIV positive and HIV negative groups, facilitating appropriate tailored approaches to each sub-group. The benefits of these would include policy changes and streamlined cost-effective treatment plans that will be equally beneficial to patient and the health department.

Legend for Figure 1

List of all study procedures utilised in the patient assessment

For peer review only - http://bmjopen.bmj.com/site/about/guidelines.xhtml

REFERENCES

1. Subedi S, Chalise P, Aich T, Thapa D. Psychiatric co-morbidity in HIV/AIDS: A neglected issue. Journal of Psychiatrists' Association of Nepal. 2013;2(2):6-10.

2. De Hert M, Cohen D, Bobes J, Cetkovich-Bakmas M, Leucht S, Ndetei DM, et al. Physical illness in patients with severe mental disorders. II. Barriers to care, monitoring and treatment guidelines, plus recommendations at the system and individual level. World Psychiatry. 2011;10(2):138-51.

3. Wainberg ML, Scorza P, Shultz JM, Helpman L, Mootz JJ, Johnson KA, et al. Challenges and Opportunities in Global Mental Health: A Research-to-Practice Perspective. Curr Psychiatry Rep. 2017;19(5):28-.

4. Shao Y, Williamson C. The HIV-1 epidemic: low-to middle-income countries. Cold Spring Harbor perspectives in medicine. 2012;2(3):a007187.

5. Breuer E, Myer L, Struthers H, Joska JA. HIV/AIDS and mental health research in sub-Saharan Africa: a systematic review. African Journal of AIDS Research. 2011;10(2):101-22.

6. De Ronchi D, Bellini F, Cremante G, Ujkaj M, Tarricone I, Selleri R, et al. Psychopathology of first-episode psychosis in HIV-positive persons in comparison to first-episode schizophrenia: A neglected issue. AIDS Care. 2006;18(8):872-8.

7. Mashaphu S, Mkize D. HIV seropositivity in patients with first-episode psychosis. South African Journal of Psychiatry. 2007;13(3):90-4.

8. Lundberg P, Nakasujja N, Musisi S, Thorson AE, Cantor-Graae E, Allebeck P. HIV prevalence in persons with severe mental illness in Uganda: a cross-sectional hospital-based study. International journal of mental health systems. 2013;7(1):20.

9. Mere SM, Paruk S. Chart review of human immunodeficiency virus status in patients admitted with psychosis in Durban, South Africa. South African Journal of Psychiatry. 2018;24. doi: 10.4102/sajpsychiatry.v24i0.1129

10. Dolder CR, Patterson TL, Jeste DV. HIV, psychosis and aging: past, present and future. AIDS. 2004;18:35-42.

11. Nakasujja N, Allebeck P, Agren H, Musisi S, Katabira E. Cognitive dysfunction among HIV positive and HIV negative patients with psychosis in Uganda. PLoS One. 2012;7(9): e44415.

For peer review only - http://bmjopen.bmj.com/site/about/guidelines.xhtml

12. Bendall S, Jackson HJ, Hulbert CA, McGorry PD. Childhood Trauma and Psychotic Disorders: a Systematic, Critical Review of the Evidence. Schizophrenia Bulletin. 2007;34(3):568-79.

13. Burns JK, Jhazbhay K, Esterhuizen T, Emsley R. Exposure to trauma and the clinical presentation of first-episode psychosis in South Africa. Journal of Psychiatric Research. 2011;45(2):179-84.

14. Bailey T, Alvarez-Jimenez M, Garcia-Sanchez AM, Hulbert C, Barlow E, Bendall S. Childhood Trauma Is Associated with Severity of Hallucinations and Delusions in Psychotic Disorders: A Systematic Review and Meta-Analysis. Schizophrenia Bulletin. 2018;44(5):1111-22.

15. Leserman J. Role of Depression, Stress, and Trauma in HIV Disease Progression. Psychosomatic Medicine. 2008;70(5):539-45.

16. Ethekwini district AIDS council quarter 1, 2017/2018 report

17. American Psychiatric Association. Diagnostic and Statistical Manual of Mental Disorders. Fifth Edition American Psychiatric Association; Washington, DC: 2013.

18. Sheehan DV, Lecrubier Y, Sheehan KH, Amorim P, Janavs J, Weiller E, et al. The Mini-International Neuropsychiatric Interview (M.I.N.I): The development and validation of a structured diagnostic psychiatric interview for DSM-IV and ICD-10. The Journal of Clinical Psychiatry. 1998;59(Suppl 20):22-33.

19. Murray CJ, Lopez AD, Organization WH. The global burden of disease: a comprehensive assessment of mortality and disability from diseases, injuries, and risk factors in 1990 and projected to 2020: summary: World Health Organization; 1996.

20. Bernstein DP, Stein JA, Newcomb MD, Walker E, Pogge D, Ahluvalia T, et al. Development and validation of a brief screening version of the Childhood Trauma Questionnaire. Child abuse & neglect. 2003;27(2):169-90.

21. Chouinard G, Margolese HC. Manual for the Extrapyramidal Symptom Rating Scale (ESRS). Schizophrenia Research. 2005;76(2):247-65.

22. WHOQOL Group. Development of the World Health Organization WHOQOL- quality of life assessment. Psychological medicine. 1998;28(3):551-8.

23. WHO ASSIST Working Group. The alcohol, smoking and substance involvement screening test (ASSIST): development, reliability and feasibility. Addiction. 2002;97(9):1183-94.

For peer review only - http://bmjopen.bmj.com/site/about/guidelines.xhtml

24. Joska JA, Witten J, Thomas KG, Robertson C, Casson-Crook M, Roosa H, et al. A Comparison of Five Brief Screening Tools for HIV-Associated Neurocognitive Disorders in the USA and South Africa. AIDS Behav. 2016;20(8):1621-31.

25. Kay SR, Fiszbein A, Opler LA. The Positive and Negative Syndrome Scale (PANSS) for Schizophrenia. Schizophrenia Bulletin. 1987;13(2):261-76.

26. Kroenke K, Spitzer RL, Williams JB. The PHQ-9: validity of a brief depression severity measure. Journal of general internal medicine. 2001;16(9):606-13.

27. Sacktor NC, Wong M, Nakasujja N, Skolasky RL, Selnes OA, Musisi S, et al. The International HIV Dementia Scale: a new rapid screening test for HIV dementia. AIDS. 2005;19(13):1367-74.

28. Weathers FW, Litz BT, Keane TM, Palmieri PA, Marx BP, Schnurr PP. The ptsd checklist for DSM-V-5 (pcl-5). Scale available from the National Center for PTSD at www ptsd va gov. 2013;10.

29. World Health Organization. Pathways of patients with mental disorders: A multi-centre collaborative project. World Health Organization; 1987.

For peer review only

For peer review only - http://bmjopen.bmj.com/site/about/guidelines.xhtml

Authors' Contributions

UC, VN, TN and SP conceived the study. UC, VN will collect data. AT performed the statistical analysis. SP and BC supervised the work undertaken. UC wrote the first draft. All authors have read and approved the final manuscript.

Funding

This study is funded by National Research Foundation of South Africa (Grant #: 117858) and the Society for Biological Psychiatry Research Fund. Funding (Grant #: N/A) was also received from University of KwaZulu-Natal College of Health Sciences PhD Scholarship (Grant #: N/A). The opinions and findings presented are those of the authors and not a reflection of the funders

Competing interests

None declared.

For peer review only

For peer review only - http://bmjopen.bmj.com/site/about/guidelines.xhtml

Table 1.

Objectives and measures used at each visit in HIV infected and non-infected participants

Domain	Measures	Source of measures/tool	Visit 1 Within first 6 weeks	Visit 2 & 3 At 3 and 6 months	Visit 4 At 12 months
Demographic factors	Socio-demographic data	Socio-demographic questionnaire	X		
Mental health outcome (Objective #1)	Psychosis	Psychiatric history and MINI	X		
	DUP & pathways to care	WHO Encounter	X		
	Severity and nature of psychosis symptoms	PANSS	X	X	X
	Depression	PHQ9	X	X	X
	Trauma	CTQ	X		
		PCL		X	
	PTSD	Clinician administered PTSD scale for DSM-V (CAPS 5)		X	
	Motor symptoms	ESRS	X	X	X
	HIV associated cognitive deficit screen	CAT-Rapid and IHDS	X	X	X
	Social cognition	Neuropsychological battery		X (at 3 months only)	X
	Substance use	WHO ASSIST	X	X	X
	Quality of life	WHO-QOL BREF	X	X	X
	Biochemical haematological and radiological tests	From Hospital Records: Full blood count, liver Function test, syphilis and Hepatitis test. Cerebrospinal fluid biochemistry and neuroimaging if available.			
Clinical markers of HIV (Objective #2)	HIV status	Confirmatory HIV ELISA			
	HIV marker	VL if HIV positive	If applicable	If applicable	If applicable
	HIV marker	CD4 if HIV positive	X		

For peer review only

Table 2.

Cognitive test battery and domains to be tested

Domains	Test
Motor skills	Pegboard, Grip strength
Visuospatial functioning	WAIS R Block Design, Digit symbol, and<br>Rey-Osterrieth Rey Complex Figure (RCF) - Copy
Verbal Memory	Rey Auditory Verbal Learning Test (RAVLT)
Non-Verbal Memory	Rey-Osterrieth Rey Complex Figure (RCF) - Recall
Problem solving/Reasoning	WAIS R Language test
Language	Rey Auditory Verbal Learning Test (RAVLT)
Word generation	Controlled Word Association Test (COWAT)<br>Animal Naming (Letter and category Cues)
Attention	WAIS R Digit Span
Executive functioning	Trail making,<br>Rey-Osterrieth Rey Complex Figure (RCF)<br>Wisconsin Card Sorting
Social cognition	Emotion perception (Faces Test),<br>Theory of mind (Eyes Test),<br>Social knowledge (Situational Features Recognition Test) (SFRT)

For peer review only

For peer review only - http://bmjopen.bmj.com/site/about/guidelines.xhtml

Figure 1.
List of all study procedures utilised in the patient assessment.

Objective 1

1. A structured socio-demographic and clinical questionnaire: age; gender; marital status; educational and employment levels; medical, psychiatric and trauma history.
2. Mini International Neuro-psychiatric Interview (MINI) version 7.02: to confirm DSM V diagnosis of psychotic disorder [18]
3. The Positive and Negative Syndrome Scale (PANSS) [24]
4. Physical examination: for weight, height and body mass index
5. Duration of Untreated Psychosis (DUP) and pathways: to care assessed with the WHO Pathways to care questionnaire, WHO Encounter [29]
6. Patient Health Questionnaire (PHQ-9) [26]
7. Cognitive screening tools: Cognitive Assessment Tool (CAT) - rapid [24] and International HIV Dementia Scale (IHDS) [27]
8. Cognitive battery (described in table 2)
9. Extrapyramidal Symptom Rating Scale (ESRS) [21]
10. Childhood trauma questionnaire Short Form (CTQ-SF) [20]
11. Posttraumatic Stress Disorder (PTSD) checklist for DSM-V (PCL-5) – Life Events Checklist (LEC)-5 and extended criterion [28]
12. WHO Quality of Life Brief Version [22]
13. WHO Alcohol, Smoking and Substance Involvement Screening Test [23]

Objective 2

14. HIV ELISA testing for all participants
15. HIV markers- CD4 count and viral load for PLWHIV at baseline, 3, 6- and 12-month visits.

215x368mm (200 x 200 DPI)

BMJ Open

Investigating the impact of HIV on patients with first episode psychosis: a study protocol for a longitudinal cohort study

Journal:	*BMJ Open*
Manuscript ID	bmjopen-2020-046593.R1
Article Type:	Protocol
Date Submitted by the Author:	29-Mar-2021
Complete List of Authors:	Chhagan, Usha; University of KwaZulu-Natal,<br>Ntlantsana, Vuyokazi; University of KwaZulu-Natal<br>Tomita, Andrew; University of KwaZulu-Natal College of Health Sciences,<br>Naidu, Thirusha; University of KwaZulu-Natal<br>Chiliza, Bonginkosi; University of KwaZulu-Natal, School of Clinical Medicine<br>Paruk, Saeeda; University of KwaZulu-Natal
**Primary Subject Heading**:	Mental health
Secondary Subject Heading:	HIV/AIDS
Keywords:	Schizophrenia & psychotic disorders < PSYCHIATRY, HIV & AIDS < INFECTIOUS DISEASES, MENTAL HEALTH

SCHOLARONE™
Manuscripts

I, the Submitting Author has the right to grant and does grant on behalf of all authors of the Work (as defined in the below author licence), an exclusive licence and/or a non-exclusive licence for contributions from authors who are: i) UK Crown employees; ii) where BMJ has agreed a CC-BY licence shall apply, and/or iii) in accordance with the terms applicable for US Federal Government officers or employees acting as part of their official duties; on a worldwide, perpetual, irrevocable, royalty-free basis to BMJ Publishing Group Ltd ("BMJ") its licensees and where the relevant Journal is co-owned by BMJ to the co-owners of the Journal, to publish the Work in this journal and any other BMJ products and to exploit all rights, as set out in our licence.

The Submitting Author accepts and understands that any supply made under these terms is made by BMJ to the Submitting Author unless you are acting as an employee on behalf of your employer or a postgraduate student of an affiliated institution which is paying any applicable article publishing charge ("APC") for Open Access articles. Where the Submitting Author wishes to make the Work available on an Open Access basis (and intends to pay the relevant APC), the terms of reuse of such Open Access shall be governed by a Creative Commons licence – details of these licences and which Creative Commons licence will apply to this Work are set out in our licence referred to above.

Other than as permitted in any relevant BMJ Author's Self Archiving Policies, I confirm this Work has not been accepted for publication elsewhere, is not being considered for publication elsewhere and does not duplicate material already published. I confirm all authors consent to publication of this Work and authorise the granting of this licence.

For peer review only

Investigating the impact of HIV on patients with first episode psychosis: a study protocol for a longitudinal cohort study

Usha Chhagan[1], Vuyokazi Ntlantsana[1], Andrew Tomita[2,3], Thirusha Naidu[4], Bonginkosi Chiliza[1], Saeeda Paruk[1]

[1] Discipline of Psychiatry, Nelson R Mandela School of Medicine, University of KwaZulu-Natal, Durban, South Africa

[2] KwaZulu-Natal Research Innovation and Sequencing Platform (KRISP), College of Health Sciences, University of KwaZulu-Natal, Durban, South Africa

[3] Centre for Rural Health, School of Nursing and Public Health, College of Health Sciences, University of KwaZulu-Natal, Durban, South Africa

[4] Department of Behavioural Medicine, University of KwaZulu-Natal, Durban, South Africa

Corresponding author
Dr Usha Chhagan

Author affiliations
Usha Chhagan, uchhagan@hotmail.com, Discipline of Psychiatry, Nelson R Mandela School of Medicine, University of KwaZulu-Natal, Durban, South Africa. +27 031-260-4321.

Vuyokazi Ntlantsana, Ntlantsanav@ukzn.ac.za, Discipline of Psychiatry, Nelson R Mandela School of Medicine, University of KwaZulu-Natal, Durban, South Africa. +27 031-260-4321.

Andrew Tomita, Tomita@ukzn.ac.za, KwaZulu-Natal Research Innovation and Sequencing Platform (KRISP), College of Health Sciences, University of KwaZulu-Natal, Durban, South Africa. Centre for Rural Health, School of Nursing and Public Health, College of Health Sciences, University of KwaZulu-Natal, Durban, South Africa. +27 031-260-4321.

Thirusha Naidu, naidut10@ukzn.ac.za, Department of Behavioural Medicine, University of KwaZulu-Natal, Durban, South Africa. +27 031-260-4321.

Bonginkosi Chiliza, ChilizaB@ukzn.ac.za, Discipline of Psychiatry, Nelson R Mandela School of Medicine, University of KwaZulu-Natal, Durban, South Africa.

Saeeda Paruk, saeedaparuk@gmail.com, Discipline of Psychiatry, Nelson R Mandela School of Medicine, University of KwaZulu-Natal, Durban, South Africa. +27 031-260-4321.

For peer review only

Investigating the impact of HIV on patients with first episode psychosis: a study protocol for a longitudinal cohort study

ABSTRACT

Introduction: South Africa (SA) has a high HIV prevalence and limited mental health care resources. Neuropsychiatric complications such as psychosis onset in people living with HIV (PLWHIV) remains poorly understood. The study aims to compare the socio-demographic, clinical, substance use, cognitive and trauma profile of PLWHIV presenting with first episode psychosis (FEP) to those with the condition but without HIV.

Methods and Analysis: This study will compare presentation, course, and outcome of a cohort of PLWHIV and FEP with a control group recruited over a 3-year period. We will prospectively test the hypothesis that the 2 groups are socio-demographically, clinically and cognitively distinct at illness presentation, with higher trauma burden and poorer outcomes in those with the dual burden of HIV and FEP. FEP participants, confirmed by a structured neuropsychiatric interview, will have their socio-demographic, psychosis, mood, motor, trauma, and substance use variables assessed. A neuropsychological battery will be completed to assess cognition, while quality of life, psychotic symptoms and HIV markers will be measured at 3, 6 and 12 months.

Ethics and dissemination: The study protocol has been reviewed and ethics approval obtained from the Biomedical Research Ethics Committee (BC 571/18) of the University of KwaZulu-Natal. The results from this investigation will be actively disseminated through peer-reviewed journal publications and conference presentations.

Keywords: Mental Health; HIV; Psychosis; Africa.

For peer review only - http://bmjopen.bmj.com/site/about/guidelines.xhtml

Strengths and limitations of this study

- This study will be among the first to examine the interacting roles of HIV, substance use and trauma in a FEP study in Africa.
- The study may provide valuable insight into the long-term impact of HIV infection on psychosis presentation which is poorly understood in low- and middle-income countries (LMIC).
- Findings from this study will contribute towards baseline data to guide future studies of patients with FEP.
- The study is hospitals-based, and this may introduce sample bias and limit generalizability to community samples.
- Randomized controlled trial (RCT) design is not possible given that study monitors disease progression.

For peer review only

INTRODUCTION

Psychiatric illness in the presence of Human Immunodeficiency virus (HIV) and Acquired Immunodeficiency Syndrome (AIDS) has been linked to negative health behaviours and poorer clinical outcomes[1]. The prevalence of HIV among those with serious mental illness (SMI) ranges from 1 to 24%[2]. Left untreated, mental disorders in people living with HIV (PLWHIV) result in poorer quality of life, greater interpersonal difficulties, increased suicide risk and poor adherence to antiretroviral therapy.

Psychotic disorders are associated with a significant burden of disease, with affected persons often relying on limited public sector mental health resources in LMICs[3]. There is a lack of research on HIV and psychosis in LMICs, particularly sub-Saharan Africa (SSA), despite this region accounting for more than two-thirds of the world's HIV-positive population[4]. While research from high-income countries has demonstrated that there is a bi-directional relationship between HIV/AIDS and mental illness, there may be differences in the way this relationship is expressed in SSA[5]. There is much needed cross-cultural insight into the relationship between HIV/AIDS and psychosis. Culture may influence the patients' perception and experience of psychosis as well as the clinical presentation. This highlights the need to explore the impact of culture on psychosis[6]. Studies have also shown variations in the socio-demographic and clinical profile of those with dual burden of HIV and first episode psychosis compared to those with psychosis only[7].

Socio-demographic profile of PLWHIV and first episode psychosis

There is limited literature on the association of HIV and FEP, with previous studies mainly describing HIV prevalence rates or clinical profiles[8]. A Ugandan study by Lundberg and colleagues[9] found that HIV positive patients with mental illness were more likely to be females and older (40-49 years). A KwaZulu-Natal (KZN) Province, SA, study of patients with FEP reported a higher HIV prevalence in less educated individuals, however, this was a small sample[8]. In a more recent chart review of patients with psychosis admitted to a psychiatric hospital in SA, HIV-infected patients with psychotic disorders were more likely to be female (74.0%), younger than 50 years (94.0%) and less likely to have secondary education than HIV-negative patients with psychotic disorders (56.0% vs. 72.0%)[10].

For peer review only - http://bmjopen.bmj.com/site/about/guidelines.xhtml

PLWHIV and psychosis symptomatology

In a review of the literature, grandiose, persecutory, and somatic delusions were the most common psychotic symptoms, followed by hallucinations and mood symptoms in PLWHIV with FEP[11]. An Italian study found that HIV infection increased the severity of symptoms (more frequently paranoid delusions), was associated with a greater impairment in attention and concentration, and had decreased depressive symptoms[7]. In addition, PLWHIV and psychosis are more likely to be diagnosed as having a psychosis secondary to another medical condition than a primary psychotic disorder, have more comorbid medical disorders and increased haematological test abnormalities[10]. A study in Gauteng province, SA, found that amongst PLWHIV presenting to psychiatry services, 23 individuals were diagnosed with psychosis due to another medical condition whilst only two were diagnosed with schizophrenia[12]. However, there is extremely limited literature on HIV and psychosis symptomatology, particularly from Africa[8,13]. This suggests a hiatus in the current literature on understanding the nature and severity of psychotic symptoms in PLWHIV, particularly in the African context. There is a need for systematic studies of larger cohorts with standardised tools to better understand how HIV may potentially influence psychosis presentation and course, as well as HIV disease outcomes.

PLWHIV and FEP and cognition

Cognitive impairment is well established in both psychotic disorders and HIV infection, being exacerbated in psychotic patients with HIV[13]. Being HIV positive is traditionally associated with a sub-cortical cognitive impairment, the prevalence of cognitive deficits being reported to range from 4% - 99% in a systematic review of HIV research in SSA[5].

De Ronchi[7] described cognitive screen deficits in a sample of 22 FEP patients living with HIV in Italy. Participants had impaired attention and concentration on the Mini Mental State Examination (MMSE) but no other differences on cognitive impairment. A study of cognitive dysfunction among 156 HIV infected and 322 HIV non-infected patients with psychosis in Uganda assessed them with the MMSE and a neuropsychological battery. They found that PLWHIV and psychosis were more cognitively impaired than HIV negative psychotic patients at baseline, and although there was some improvement with treatment, they remained more impaired at six months, suggesting that HIV worsened cognitive dysfunction in psychosis[13]. While earlier studies have suggested that psychosis and impairment in cognition were later manifestations of HIV, the Ugandan study demonstrated that both these

For peer review only - http://bmjopen.bmj.com/site/about/guidelines.xhtml

conditions occur early, as shown by the average CD4 count of 305 cell/uL and the intermediate World Health Organization (WHO) staging of disease presentation. In addition, impairment in cognition also remained, despite an improvement in the psychotic symptoms[13]. Further research is warranted to establish the impact of HIV and psychosis on cognition, and whether HIV related cognitive changes are associated with increased risk for psychosis or modifies psychosis onset or course.

PLWHIV and FEP and substance use

Studies traditionally suggest an association between substance use in FEP patients and HIV infection[1]. Substance use patterns vary across the different regions of SA. This may be related to both ethnic and socio-economic differences[14]. Davis et al. confirmed both the growing pattern of substance use as well as the trend of psychiatric comorbidity in KwaZulu-Natal (KZN). The rates of substance use in this province were also significantly higher than that reported in studies of the general population in SA[14]. Another study of patients with FEP in KZN, SA, reported lower rates of self-reported substance use among HIV positive then HIV negative patients with mental illness[8], which was further supported by a retrospective chart review of patients with psychosis[10]. This may suggest that either substance use/misuse may not be a significant factor in HIV transmission in the KZN context, or that the study results were limited by under-reporting or the small sample size. Thus, while the international literature suggests that PLWHIV and mental illness may be vulnerable to substance use, this has not been borne out in the local KZN studies, and warrants further research.

PLWHIV and FEP and Trauma

Trauma, particularly early life trauma, is also recognised as an environmental risk factor for psychosis[15]. A South African study investigated the association between a history of traumatic experiences and the clinical features of FEP. Duration of untreated psychosis, age of and symptoms at onset were assessed in 54 psychotic patients, while 22% were HIV infected. Almost half of the study subjects had witnessed (49%) or directly experienced serious physical assault (45%), both events being associated with positive psychotic symptoms[16].

For peer review only - http://bmjopen.bmj.com/site/about/guidelines.xhtml

The trauma experienced may influence psychosis and HIV outcomes, as found in a systematic review, that hallucinations and delusions were more severe in people with a history of childhood trauma[17]. In addition, Leserman[18] reported on the negative association of HIV, stress, and depression on the course of HIV in terms of decreasing the CD4 lymphocytes, increasing the viral load and having greater risk for premature mortality.

Exposure to violence and resultant trauma is of major public health concern. In SA, the mortality rate secondary to violence was estimated at 73/100,000[19,20]. Whilst much research on early trauma has been conducted in developed countries, there is a need to examine the association of trauma and FEP in developing countries[19,20,21].

Course of comorbid HIV and psychosis

PLWHIV and psychosis are more likely to develop comorbid medical disorders, experience side effects of antipsychotic medication, develop metabolic syndrome secondary to ART, have longer length of hospital stay and readmission rates, and poorer quality of life[1,10]. However, this has not been tested prospectively in the FEP patients in the African context with the recent wide availability of ART.

Aims

The aim of the study is to investigate trends in clinical, substance use, cognitive and trauma presentations in individuals experiencing first episode of psychosis over time, and to compare presentation, course and 12-month outcomes between those with and without HIV.

METHODS

Patients referred to the participating psychiatry units will be screened by the respective treating doctors for eligibility for recruitment into the study. The patients with FEP must fulfil the inclusion criteria and will be referred to the designated investigator at each unit. Once written informed consent has been obtained, the protocol for data capture will be followed, as per the list outlined in Table 1 and Figure 1, the same protocol being followed for out- and in-patients. The clinical examination and psychiatric tests, which take approximately two hours, will be undertaken by the investigators who are all medical practitioners. Patient baseline assessments will be undertaken within six weeks of treatment during their outpatient visit or following admission to the respective units at baseline. A physical examination, body mass index and waist circumference measure will also be

For peer review only - http://bmjopen.bmj.com/site/about/guidelines.xhtml

obtained by a medical practitioner. Where possible, collateral information from a next of kin, with the participant's consent, will also be sought. The follow-up visits will be done at three, six and 12 months as outpatients, and the participants will be reimbursed for travel costs. Attempts will be made to coincide each of these with scheduled clinic follow-up visits and will be performed by the same initial interviewer at each psychiatry outpatient department.

The cognitive battery assessment, which takes approximately 2 hours, will be administered by a clinical psychologist, at the three- and 12-month visits. Translation services will also be available for patients who require isiZulu translation during the interviews.

Following collating the data for steps 1-13 in Figure 1 and Table 2 [22,23,24,25,26,27,28,29,30,31,32,33], we will perform steps 14 and 15 after voluntary counselling and testing. The required HIV tests, CD4 count and viral load if HIV positive, as per the protocol, will be taken by a medical practitioner at each site. HIV testing will be carried out at baseline for all participants as well as at each visit for HIV negative participants. A patient with a positive HIV test will be referred to the HIV clinic at the site for further management. Other investigations, such as lipid profile, which are part of routine care, will be captured from the clinical records.

We are keen to explore impact of pathways of care on sociodemographic and clinical variables. Rehabilitation programs are limited as many sites lack a multi- disciplinary team to support this, however standard of care includes medication, supportive therapy, psycho-education, referral to psychology if indicated, and management of comorbid substance use.

Study design and setting

The comparative cohort study will be in keeping with a quantitative, descriptive and longitudinal 12-month design of adult patients presenting with FEP that are either HIV infected or non-infected. The study will take place in the eThekwini District, one of 11 in KZN Province, with an estimated population of 3,442,361 people. The district has a high HIV burden, with an estimated 650 000 people living with the virus in 2018[34]. In addition, there are only five hospitals in the municipality with psychiatric services managed by a psychiatrist, at which the study will be conducted. The hospitals selected are the four general hospitals with specialist psychiatric services situated in eThekwini: Addington, King Edward VIII, Prince Mshiyeni and RK Khan Hospitals, and at King Dinuzulu Hospital, which serves

For peer review only - http://bmjopen.bmj.com/site/about/guidelines.xhtml

as the psychiatric hospital for the area. All hospitals have in- and out-patient psychiatric services and receive referrals from within the district as well as from urban and rural areas as far south as the Eastern Cape Province and northern KZN.

Eligibility criteria

Inclusion criteria

Male and female patients will be included if they are in or out-patients, aged 18-45 years, have a first presentation to mental health care services for FEP meeting Diagnostic and Statistical Manual (DSM)-V criteria[35] (American Psychiatric Association (APA) 2013) for a psychotic disorder, are neuroleptic naïve or minimally treated (maximum 6 weeks of psychotropic treatment in this first episode), and provide consent to participate.

Exclusion criteria

Patients will be excluded if they have been prescribed antipsychotics in the past or have had previous psychotic episode, an intellectual disability (assessed clinically or by chart review) or other significant general medical conditions that maybe associated with FEP e.g., epilepsy or syphilis, have less than seven years of formal schooling and refuse consent.

Participant selection and sampling strategy

All adult in-or out patients with FEP meeting the inclusion criteria will be recruited to the study over a 3-year period. The sample size calculation was based on a log rank test group. In order to achieve an 80% power to detect an odds ratio of 1.857 (to maximise variability), in a design with three repeated measurements having a compound symmetry covariance structure when the proportion from group 2 is 0.500, the correlation between observations on the same subject is 0.500, and the alpha (significance) level 0.05 or 5%, a sample of 130 PLWHIV and FEP and 130 HIV negative with FEP will be required. In addition, this sample size accounts for and permits a 10% dropout rate over an anticipated 36-month accrual with a minimum follow-up of 12 months.

Data collection

The study will utilize a clinical interview, physical examination, several psychiatric tools, and HIV related haematological tests to measure variables. Baseline measures will be done within six weeks of first presentation and as soon as the participant is able to consent. Participants need to have responded to antipsychotic medication and will be assessed to have the capacity

For peer review only - http://bmjopen.bmj.com/site/about/guidelines.xhtml

to consent by a treating and research doctor. The study (HIV+) and control groups (HIV-) will receive the same baseline assessments and follow-up at three, six and 12 months, which are summarised in Table 1. The HIV negative group will have HIV testing at each visit to re-confirm HIV status. The study procedures, with instruments[36,37,38,39,40,41,42,43,44,45,46], that will be used are shown in the information Figure 1. Data will be collected manually and captured electronically.

Clinical researchers with a medical background will administer the mental health diagnostic - assessment, the MINI 7.02, Psychotic Disorders version, DSM-V[36] and diagnostic interview face-to-face, and will be supported by a trained research assistant who will facilitate the self-report questionnaires. All study personnel will be trained on initiation of the study with an annual refresher training on the different tools by psychiatrists with research and clinical experience. A medical doctor will conduct the clinical assessments, collect the biological specimens (Table 1), and conduct the HIV counselling and testing. We will use an HIV COBAS COMBI test to detect both HIV antibodies and p24 antigen. Standard pre-test and post-test counselling will be delivered in a culturally sensitive manner, while the CD4 count and viral loads will be measured at every visit among PLWHIV.

Participants will be given a return research appointment date and telephonic contact will be maintained between interviews. The study team (research assistant and clinician) will contact participants to remind them of appointments to limit their withdrawing from the study. For participants who do not arrive for their appointments, the research assistant will contact the patient to reschedule an appointment in the same week.

Data management and analysis

All participants will be allocated a participant identification number (PID) code at enrolment, which will be used on samples and documents over the 12-month study period. Data will be downloaded to a secure server and kept in a password-protected system. Laboratory results will be merged into the main dataset using the unique PID.

Data was will be entered into SPSS version 24 and the analyses conducted using STATA 15. The socio-demographic and clinical characteristics of participants will be summarized using means and standard deviation (SD) continuous variables. For categorical variables, proportion (%) will be reported. Significant associations in contingency tables (cross

For peer review only

For peer review only - http://bmjopen.bmj.com/site/about/guidelines.xhtml

tabulations) will be assessed using the standard Pearson's chi-square (χ^2) test. An independent samples t-test will be used for comparing differences in continuous variables between two groups. If there are more than three groups, we will utilise ANOVA methods. A p-value less than 0.05 will be regarded as statistically significant. In addition, we may also apply growth mixture modelling to observe whether subsets of individuals follow distinct trajectories over time.

Patient and public involvement

No patients or members of the public were involved in the design, analysis or reporting of this current investigation.

Ethics and dissemination

Ethical approval to conduct the study was obtained from the Biomedical Research Ethics Committee of the University of KwaZulu-Natal (UKZN). The study will be conducted in accordance with South African Department of Health Research Ethics guidelines (2015) as well as the UKZN policy on Research Ethics. Recruitment of study participants will commence as full ethics approval was received from Biomedical Research Ethics Committee. No other independent ethics review was required.

All participants fulfilling the inclusion criteria will be selected for the study, from whom written, informed consent will be obtained. All potential participants will have the opportunity to participate, which will be made known to them in the information sheet. Where participants are unable to consent independently, adequate steps will be taken to ensure that legally acceptable proxy consent is obtained. All participants testing HIV positive at any point in the study will be referred for ART and support.

The results from this investigation will be actively disseminated through peer-reviewed journal publications and conference presentations. Upon completion of the peer-review process, we will provide feedback to clinicians at Department of Health for further discussion about enhanced treatment algorithm.

DISCUSSION

Potential risks

For peer review only - http://bmjopen.bmj.com/site/about/guidelines.xhtml

There will be minimal risk or discomfort to participants, with one test requiring bloods to be taken. The only active involvement of the participants is for them to provide verbal responses to assessment interviews and scales utilized for data collection. We acknowledge the potential risk of psychological distress to patients, as they may be directly affected by sensitive questions e.g., relating to trauma during the administration of the psychiatric scales. The study has a distress plan to support such patients, which entails referral to treating team if there is concern about relapse or danger to self or others. The sites have specialist psychiatric services, both inpatient and outpatient, with a team available to attend to any adverse event that may arise during the interview process.

Methodological challenges and study limitations

Several limitations may affect the study, including that it is hospital-based, which may introduce sample bias and limit the generalizability to community samples. Another limitation is the use of tools that have limited validity in the local South African context, as they have not been used previously. Language and cultural barriers in communication will be addressed by an available isiZulu translator. A further limitation of the observational nature of the study is the attendant lack of standardisation of treatment, for which will follow the Essential Drug List (EDL) for hospital level adults (Department of Health, 2015). Although patients will follow the same algorithm, they may be maintained on different medication, depending on the individual patient treatment response. The study will however provide an adequate sample representing the clinical profile of patients with FEP and PLWHIV in a resource-constrained setting.

Study progress and challenges

A challenge we anticipate is the loss of participant follow-up, which is generally a common failing in managing psychiatric patients, particularly in LMIC settings with challenging psychosocial circumstances. The three-, six- and 12-month follow-up appointments may remain difficult to sustain, despite supporting participants with travel costs for visits. Maintaining follow-up visits is a challenge we anticipated and for which we have a contingency strategy, this being that a research assistant will have regular communication with the recruited participants, confirming appointment dates, sending reminders, and facilitating travel arrangements. Participants seroconverting to HIV positive status during the study will be referred to antiretroviral services for care but continue follow-up assessments, noting the change in their HIV status.

For peer review only - http://bmjopen.bmj.com/site/about/guidelines.xhtml
For peer review only

Study Significance

We postulate that the course and outcome of HIV psychosis differs from that in an HIV negative individual, and that this study may provide valuable insight into the impact of HIV infection on psychosis presentation and outcomes. Longitudinal comparative cohort studies of this nature are limited and have not been conducted locally in one of the highest HIV prevalence settings in Africa, hence the data will serve to introduce new knowledge on the topic, as well as form the baseline for future research and treatment. A clearer understanding of the association between HIV and psychosis may provide better insight into HIV as a neurotropic virus. The findings of this study will contribute towards improving the care and management of patients with FEP and HIV.

The World Health Organization reports that in 2020, psychiatric conditions are ranked the second leading cause of global disease burden after ischemic heart disease[47]. In the South African context, this is even more pronounced by the additional burden of HIV infection and AIDS. With an approximate overall HIV prevalence rate of 11.2% (6.19 million) and an estimated 16.6% of the population (adults aged 15-49 years) being HIV positive, SA is in urgent need of interventions to address the related adverse consequences. Psychotic disorders are associated with a significant burden of disease and often utilize the limited mental health resources. Understanding the association between HIV and psychosis in the era of antiretroviral treatment is thus an urgent priority. Utilizing the HIV psychosis as a potential model for schizophrenia may also improve our understanding of its neurobiology. The new knowledge, generated through this novel study, will enable an understanding of the interplay between HIV and psychosis at a fundamental level, centred at the international epicentre of the HIV pandemic.

In addition, in SA, substance use and trauma remain critical public health challenges that need to be further explored. Thus, the study of the bidirectional associations between HIV, substance use and traumatic experiences, psychosis onset and presentation in a low- and middle-income setting is essential to better guide the strategic use of their limited resources. Early insights into the role of substance use and trauma in HIV infection and psychosis onset could be used to delay, modify, or even prevent the onset of HIV psychosis. Furthermore, the interplay of these extraneous factors in genetically predisposed individuals has not been explored. This study will allow us to begin to understand the contribution of HIV to

For peer review only - http://bmjopen.bmj.com/site/about/guidelines.xhtml

psychosis onset, increase awareness of the need for screening and care in a highly vulnerable group, while building research capacity.

In LMIC, 11.1% of the total burden of disease are attributable to mental disorders[21]. In addition to the high prevalence and morbidity associated with mental disorders, it has been shown that cultural influences impact significantly on and contribute to the risk for mental and other health concerns in developing countries. The results of this study will contribute to the development of management (investigation and treatment) algorithms in the South African resource constrained health departments. Reference will be made to the differences identified between the HIV positive and HIV negative groups, facilitating appropriate tailored approaches to each sub-group. The benefits of these would include policy changes and streamlined cost-effective treatment plans that will be equally beneficial to patient and the health department.

Legend for Figure 1

List of all study procedures utilised in the patient assessment

Contributorship statement

UC, VN, TN and SP conceived the study. UC, VN will collect data. AT performed the statistical analysis. SP and BC supervised the work undertaken. UC wrote the first draft. All authors have read and approved the final manuscript.

Competing interests

None declared.

Funding

This study is funded by National Research Foundation of South Africa (Grant #: 117858), South African Research Council SIR grant and a start-up grant from the Society for Biological Psychiatry Research Fund. Funding (Grant #: N/A) was also received from University of KwaZulu-Natal College of Health Sciences PhD Scholarship (Grant #: N/A). Andrew Tomita was funded by UK Global Challenge Research Fund (MR/T029803/1). The opinions and findings presented are those of the authors and not a reflection of the funders.

For peer review only - http://bmjopen.bmj.com/site/about/guidelines.xhtml

REFERENCES

1. Subedi S, Chalise P, Aich T, Thapa D. Psychiatric co-morbidity in HIV/AIDS: A neglected issue. Journal of Psychiatrists' Association of Nepal. 2013;2(2):6-10.

2. De Hert M, Cohen D, Bobes J, Cetkovich-Bakmas M, Leucht S, Ndetei DM, et al. Physical illness in patients with severe mental disorders. II. Barriers to care, monitoring and treatment guidelines, plus recommendations at the system and individual level. World Psychiatry. 2011;10(2):138-51.

3. Wainberg ML, Scorza P, Shultz JM, Helpman L, Mootz JJ, Johnson KA, et al. Challenges and opportunities in global mental health: a research-to-practice perspective. Current psychiatry reports. 2017;19(5):28.

4. Shao Y, Williamson C. The HIV-1 epidemic: low-to middle-income countries. Cold Spring Harbor perspectives in medicine. 2012;2(3):a007187.

5. Breuer E, Myer L, Struthers H, Joska JA. HIV/AIDS and mental health research in sub-Saharan Africa: a systematic review. African Journal of AIDS Research. 2011;10(2):101-22.

6. Vermeiden M, Janssens M, Thewissen V, Akinsola E, Peeters S, Reijnders J, et al. Cultural differences in positive psychotic experiences assessed with the Community Assessment of Psychic Experiences-42 (CAPE-42): a comparison of student populations in the Netherlands, Nigeria and Norway. BMC psychiatry. 2019;19(1):1-15.

7. De Ronchi D, Bellini F, Cremante G, Ujkaj M, Tarricone I, Selleri R, et al. Psychopathology of first-episode psychosis in HIV-positive persons in comparison to first-episode schizophrenia: A neglected issue. AIDS Care. 2006;18(8):872-8.

8. Mashaphu S, Mkize D. HIV seropositivity in patients with first-episode psychosis. South African Journal of Psychiatry. 2007;13(3):90-4.

9. Lundberg P, Nakasujja N, Musisi S, Thorson AE, Cantor-Graae E, Allebeck P. HIV prevalence in persons with severe mental illness in Uganda: a cross-sectional hospital-based study. International journal of mental health systems. 2013;7(1):20.

For peer review only - http://bmjopen.bmj.com/site/about/guidelines.xhtml

10. Mere SM, Paruk S. Chart review of human immunodeficiency virus status in patients admitted with psychosis in Durban, South Africa. South African Journal of Psychiatry. 2018;24.

11. Dolder CR, Patterson TL, Jeste DV. HIV, psychosis and aging: past, present and future. AIDS. 2004;18:35-42.

12. Van Rensburg BJ, Bracken C. Acute psychiatric in-patients tested for HIV status: a clinical profile. South African Journal of Psychiatry. 2007;10(1).

13. Nakasujja N, Allebeck P, Agren H, Musisi S, Katabira E. Cognitive dysfunction among HIV positive and HIV negative patients with psychosis in Uganda. PLoS One. 2012;7(9): e44415.

14. Davis GP, Tomita A, Baumgartner JN, Mtshemla S, Nene S, King H, et al. Substance use and duration of untreated psychosis in KwaZulu-Natal, South Africa. South African Journal of Psychiatry. 2016;22(1).

15. Bendall S, Jackson HJ, Hulbert CA, McGorry PD. Childhood Trauma and Psychotic Disorders: a Systematic, Critical Review of the Evidence. Schizophrenia Bulletin. 2007;34(3):568-79.

16. Burns JK, Jhazbhay K, Esterhuizen T, Emsley R. Exposure to trauma and the clinical presentation of first-episode psychosis in South Africa. Journal of Psychiatric Research. 2011;45(2):179-84.

17. Bailey T, Alvarez-Jimenez M, Garcia-Sanchez AM, Hulbert C, Barlow E, Bendall S. Childhood Trauma Is Associated with Severity of Hallucinations and Delusions in Psychotic Disorders: A Systematic Review and Meta-Analysis. Schizophrenia Bulletin. 2018;44(5):1111-22.

18. Leserman J. Role of Depression, Stress, and Trauma in HIV Disease Progression. Psychosomatic Medicine. 2008;70(5):539-45.

For peer review only

19. Doolan K, Ehrlich R, Myer L. Experience of violence and socioeconomic position in South Africa: a national study. Plos One. 2007;2(12):e1290.

20. Matzopoulos R, Norman R, Bradshaw D. The burden of injury in South Africa: Fatal injury trends and international comparisons. Tygerberg: Medical Research Council-University of South Africa, Crime, Violence and Injury Lead Programme. 2004.

21. Patel V. Mental health in low-and middle-income countries. British medical bulletin. 2007;81(1):81-96.

22. Podell K. Hand Dynamometer. In: Kreutzer JS, DeLuca J, Caplan B, editors. Encyclopedia of Clinical Neuropsychology. New York, NY: Springer New York; 2011. p. 1208-9.

23. Merker B, Podell K. Grooved Pegboard Test. In: Kreutzer JS, DeLuca J, Caplan B, editors. Encyclopedia of Clinical Neuropsychology. New York, NY: Springer New York; 2011. p. 1176-8.

24. Wechsler D. Wechsler adult intelligence scale-revised (WAIS-R): Psychological Corporation; 1981.

25. McKinlay A. Rey Complex Figure Test. In: Goldstein S, Naglieri JA, editors. Encyclopedia of Child Behavior and Development. Boston, MA: Springer US; 2011. p. 1264-5.

26. Bean J. Rey Auditory Verbal Learning Test, Rey AVLT. In: Kreutzer JS, DeLuca J, Caplan B, editors. Encyclopedia of Clinical Neuropsychology. New York, NY: Springer New York; 2011. p. 2174-5.

27. Patterson J. Controlled Oral Word Association Test. In: Kreutzer JS, DeLuca J, Caplan B, editors. Encyclopedia of Clinical Neuropsychology. New York, NY: Springer New York; 2011. p. 703-6.

For peer review only

For peer review only - http://bmjopen.bmj.com/site/about/guidelines.xhtml

28. Patterson J. Verbal Fluency. In: Kreutzer JS, DeLuca J, Caplan B, editors. Encyclopedia of Clinical Neuropsychology. New York, NY: Springer New York; 2011. p. 2603-6.

29. Bucks RS. Trail-Making Test. In: Gellman MD, Turner JR, editors. Encyclopedia of Behavioral Medicine. New York, NY: Springer New York; 2013. p. 1986-7.

30. Jones CRG. Wisconsin Card Sorting Test (WCST). In: Volkmar FR, editor. Encyclopedia of Autism Spectrum Disorders. New York, NY: Springer New York; 2013. p. 3389-91.

31. Bänziger T. Measuring Emotion Recognition Ability. In: Michalos AC, editor. Encyclopedia of Quality of Life and Well-Being Research. Dordrecht: Springer Netherlands; 2014. p. 3934-41.

32. Baron-Cohen S. Theory of mind and autism: A review. International review of research in mental retardation. 2000;23:169-84.

33. Corrigan PW, Green MF. The Situational Feature Recognition Test: A measure of schema comprehension for schizophrenia. International Journal of Methods in Psychiatric Research. 1993.

34. Ethekwini district AIDS council quarter 1, 2017/2018 report.

35. American Psychiatric Association. Diagnostic and Statistical Manual of Mental Disorders. Fifth Edition American Psychiatric Association; Washington, DC: 2013.

36. Sheehan DV, Lecrubier Y, Sheehan KH, Amorim P, Janavs J, Weiller E, et al. The Mini-International Neuropsychiatric Interview (M.I.N.I): The development and validation of a structured diagnostic psychiatric interview for DSM-IV and ICD-10. The Journal of Clinical Psychiatry. 1998;59(Suppl 20):22-33.

37. Kay SR, Fiszbein A, Opler LA. The Positive and Negative Syndrome Scale (PANSS) for Schizophrenia. Schizophrenia Bulletin. 1987;13(2):261-76.

For peer review only

For peer review only - http://bmjopen.bmj.com/site/about/guidelines.xhtml

38. World Health Organization. Pathways of patients with mental disorders: A multi-centre collaborative project. World Health Organization; 1987.

39. Kroenke K, Spitzer RL, Williams JB. The PHQ-9: validity of a brief depression severity measure. Journal of general internal medicine. 2001;16(9):606-13.

40. Joska JA, Witten J, Thomas KG, Robertson C, Casson-Crook M, Roosa H, et al. A Comparison of Five Brief Screening Tools for HIV-Associated Neurocognitive Disorders in the USA and South Africa. AIDS Behav. 2016;20(8):1621-31.

41. Sacktor NC, Wong M, Nakasujja N, Skolasky RL, Selnes OA, Musisi S, et al. The International HIV Dementia Scale: a new rapid screening test for HIV dementia. AIDS. 2005;19(13):1367-74.

42. Chouinard G, Margolese HC. Manual for the Extrapyramidal Symptom Rating Scale (ESRS). Schizophrenia Research. 2005;76(2):247-65.

43. Bernstein DP, Stein JA, Newcomb MD, Walker E, Pogge D, Ahluvalia T, et al. Development and validation of a brief screening version of the Childhood Trauma Questionnaire. Child abuse & neglect. 2003;27(2):169-90.

44. Weathers FW, Litz BT, Keane TM, Palmieri PA, Marx BP, Schnurr PP. The ptsd checklist for DSM-V-5 (pcl-5). Scale available from the National Center for PTSD at www ptsd va gov. 2013;10.

45. WHOQOL Group. Development of the World Health Organization WHOQOL- quality of life assessment. Psychological medicine. 1998;(3):551-8.

46. WHO ASSIST Working Group.The alcohol, smoking and substance involvement screening test (ASSIST): development, reliability and feasibility. Addiction. 2002;97(9):1183-94.

47. Murray CJ, Lopez AD, Organization WH. The global burden of disease: a comprehensive assessment of mortality and disability from diseases, injuries, and risk factors in 1990 and projected to 2020: summary: World Health Organization; 1996.

For peer review only - http://bmjopen.bmj.com/site/about/guidelines.xhtml
For peer review only

For peer review only

For peer review only - http://bmjopen.bmj.com/site/about/guidelines.xhtml

Table 1.

Objectives and measures used at each visit in HIV infected and non-infected participants

Domain	Measures	Source of measures/tool	Visit 1 Within first 6 weeks	Visit 2 & 3 At 3 and 6 months	Visit 4 At 12 months
Demographic factors	Socio-demographic data	Socio-demographic questionnaire	X		
Mental health outcome (Objective #1)	Psychosis	Psychiatric history and MINI	X		
	DUP & pathways to care	WHO Encounter	X		
	Severity and nature of psychosis symptoms	PANSS	X	X	X
	Depression	PHQ9	X	X	X
	Trauma	CTQ	X		
		PCL		X	
	PTSD	Clinician administered PTSD scale for DSM-V (CAPS 5)		X	
	Motor symptoms	ESRS	X	X	X
	HIV associated cognitive deficit screen	CAT-Rapid and IHDS	X	X	X
	Social cognition	Neuropsychological battery		X (at 3 months only)	X
	Substance use	WHO ASSIST	X	X	X
	Quality of life	WHO-QOL BREF	X	X	X
	Biochemical haematological and radiological tests	From Hospital Records: Full blood count, liver Function test, syphilis and Hepatitis test. Cerebrospinal fluid biochemistry and neuroimaging if available.			
Clinical markers of HIV (Objective #2)	HIV status	Confirmatory HIV ELISA			
	HIV marker	VL if HIV positive	If applicable	If applicable	If applicable
	HIV marker	CD4 if HIV positive	X		

For peer review only - http://bmjopen.bmj.com/site/about/guidelines.xhtml

Table 2.

Cognitive test battery and domains to be tested

Domains	Test
Motor skills	Grip strength[22], Pegboard [23]
Visuospatial functioning	WAIS-R Block Design, Digit symbol[24], and Rey-Osterrieth Rey Complex Figure (RCF) – Copy[25]
Verbal Memory	Rey Auditory Verbal Learning Test (RAVLT)[26]
Non-Verbal Memory	RCF - Recall [25]
Problem solving/Reasoning	WAIS-R Language test[24]
Language	RAVLT[26]
Word generation	Controlled Word Association Test (COWAT)[27] Animal Naming (Letter and Category cues)[28]
Attention	WAIS-R Digit Span[24]
Executive functioning	Trail making[29], RCF[25], Wisconsin Card Sorting Test (WCST)[30]
Social cognition	Emotion perception (Faces Test)[31], Theory of mind (Eyes Test)[32], Social knowledge (Situational Feature Recognition Test) (SFRT)[33]

For peer review only

Figure 1.

List of all study procedures utilised in the patient assessment.

Objective 1

To compare HIV infected and HIV non-infected patients with FEP at psychosis onset in at baseline, 3-, 6- and 12-months regarding socio-demographic profile, clinical features, substance use patterns, traumatic life events and quality of life.

1. A structured socio-demographic and clinical questionnaire: age; gender; marital status; educational and employment levels; medical, psychiatric and trauma history.
2. Mini International Neuro-psychiatric Interview (MINI) version 7.02: to confirm DSM V diagnosis of psychotic disorder [36]
3. The Positive and Negative Syndrome Scale (PANSS) [37]
4. Physical examination: for weight, height and body mass index
5. Duration of Untreated Psychosis (DUP) and pathways: to care assessed with the WHO Pathways to care questionnaire, WHO Encounter [38]
6. Patient Health Questionnaire (PHQ-9) [39]
7. Cognitive screening tools: Cognitive Assessment Tool (CAT) - rapid [40] and International HIV Dementia Scale (IHDS) [41]
8. Cognitive battery (described in table 2)
9. Extrapyramidal Symptom Rating Scale (ESRS) [42]
10. Childhood trauma questionnaire Short Form (CTQ-SF) [43]
11. Posttraumatic Stress Disorder (PTSD) checklist for DSM-V (PCL-5) – Life Events Checklist (LEC)-5 and extended criterion [44]
12. WHO Quality of Life Brief Version [45]
13. WHO Alcohol, Smoking and Substance Involvement Screening Test [46]

Objective 2

To describe the association of HIV infection clinical markers (CD4 count and viral load) and clinical variables of psychosis at illness presentation, 3, 6 and 12 months.

14. HIV ELISA testing for all participants
15. HIV markers- CD4 count and viral load for PLWHIV at baseline, 3, 6- and 12-month visits.
